# Using Social Media Data to Understand the Impact of Promotional Information on Laypeople’s Discussions: A Case Study of Lynch Syndrome

**DOI:** 10.2196/jmir.9266

**Published:** 2017-12-13

**Authors:** Jiang Bian, Yunpeng Zhao, Ramzi G Salloum, Yi Guo, Mo Wang, Mattia Prosperi, Hansi Zhang, Xinsong Du, Laura J Ramirez-Diaz, Zhe He, Yuan Sun

**Affiliations:** ^1^ Department of Health Outcomes & Biomedical Informatics College of Medicine University of Florida Gainesville, FL United States; ^2^ Department of Management Warrington College of Business University of Florida Gainesville, FL United States; ^3^ Department of Epidemiology College of Public Health and Health Professions University of Florida Gainesville, FL United States; ^4^ Department of Epidemiology College of Medicine University of Florida Gainesville, FL United States; ^5^ School of Information Florida State University Tallahassee, FL United States; ^6^ School of Business Administration Zhejiang Gongshang University Hangzhou, Zhejiang China

**Keywords:** social media, Lynch syndrome, public health surveillance, sentiment analysis

## Abstract

**Background:**

Social media is being used by various stakeholders among pharmaceutical companies, government agencies, health care organizations, professionals, and news media as a way of engaging audiences to raise disease awareness and ultimately to improve public health. Nevertheless, it is unclear what effects this health information has on laypeople.

**Objective:**

This study aimed to provide a detailed examination of how promotional health information related to Lynch syndrome impacts laypeople’s discussions on a social media platform (Twitter) in terms of topic awareness and attitudes.

**Methods:**

We used topic modeling and sentiment analysis techniques on Lynch syndrome–related tweets to answer the following research questions (RQs): (1) what are the most discussed topics in Lynch syndrome–related tweets?; (2) how promotional Lynch syndrome–related information on Twitter affects laypeople’s discussions?; and (3) what impact do the Lynch syndrome awareness activities in the Colon Cancer Awareness Month and Lynch Syndrome Awareness Day have on laypeople’s discussions and their attitudes? In particular, we used a set of keywords to collect Lynch syndrome–related tweets from October 26, 2016 to August 11, 2017 (289 days) through the Twitter public search application programming interface (API). We experimented with two different classification methods to categorize tweets into the following three classes: (1) irrelevant, (2) promotional health information, and (3) laypeople’s discussions. We applied a topic modeling method to discover the themes in these Lynch syndrome–related tweets and conducted sentiment analysis on each layperson’s tweet to gauge the writer’s attitude (ie, positive, negative, and neutral) toward Lynch syndrome. The topic modeling and sentiment analysis results were elaborated to answer the three RQs.

**Results:**

Of all tweets (N=16,667), 87.38% (14,564/16,667) were related to Lynch syndrome. Of the Lynch syndrome–related tweets, 81.43% (11,860/14,564) were classified as promotional and 18.57% (2704/14,564) were classified as laypeople’s discussions. The most discussed themes were *treatment* (n=4080) and *genetic testing* (n=3073). We found that the topic distributions in laypeople’s discussions were similar to the distributions in promotional Lynch syndrome–related information. Furthermore, most people had a positive attitude when discussing Lynch syndrome. The proportion of negative tweets was 3.51%. Within each topic, *treatment* (16.67%) and *genetic testing* (5.60%) had more negative tweets compared with other topics. When comparing monthly trends, laypeople’s discussions had a strong correlation with promotional Lynch syndrome–related information on *awareness* (*r*=.98, *P*<.001), while there were moderate correlations on *screening* (*r*=.602, *P*=.05), *genetic testing* (*r*=.624, *P*=.04), *treatment* (*r*=.69, *P*=.02), and *risk* (*r*=.66, *P*=.03). We also discovered that the Colon Cancer Awareness Month (March 2017) and the Lynch Syndrome Awareness Day (March 22, 2017) had significant positive impacts on laypeople’s discussions and their attitudes.

**Conclusions:**

There is evidence that participative social media platforms, namely Twitter, offer unique opportunities to inform cancer communication surveillance and to explore the mechanisms by which these new communication media affect individual health behavior and population health.

## Introduction

In 2000, President Bill Clinton signed a White House Proclamation that March was to be designated as the Colon Cancer Awareness Month to bring attention to the second leading cause of cancer death in the United States. Lynch syndrome, also known as hereditary nonpolyposis colorectal cancer (HNPCC), is an inherited disorder that increases the risk of colon and rectum cancers, in particular, and many other types of cancer such as the stomach, liver, gallbladder ducts, small intestine, upper urinary tract, brain, and skin [1]. Lynch syndrome is the most common cause of hereditary colorectal cancer, accounting for approximately 2% to 3% of inherited colon cancer cases [2]. March 22 is recognized as the Lynch Syndrome Awareness Day by communities around the world [3].

Social media brought rapid changes to the health communication landscape. In particular, social media platforms have been used to promote healthy behavior [4], improve medical and patient education [5,6], overcome barriers in the delivery of health care [7], and address public health surveillance issues [8,9]. On one side, public health stakeholders, including health organizations, government agencies, pharmaceutical companies, news media, and advocators, use social media to broadly disseminate health information on the Internet. On the other hand, laypeople share their personal health experience, post comments, and express opinions toward specific health issues, medical products, and health care services. However, there have been very few studies on Lynch syndrome and social media. Through a PubMed search, we found only one study, where the authors asked an advocacy organization to disseminate their study information on Facebook to show the feasibility of recruiting participants with Lynch syndrome on a social media platform [10].

Twitter is a free social media platform that enables users to send and read short 140-character posts called “tweets.” Twitter analyses have been used in numerous biomedical and public health studies, with a broad range of health topics [11]. For example, Broniatowski et al have successfully used Twitter data for influenza surveillance [12]. Workewych et al hypothesized that Twitter data might be useful for understanding public perceptions and misperceptions of sport-related traumatic brain injuries [13]. Massey et al quantified human papillomavirus (HPV) vaccination communication on Twitter and used sentiment analysis to examined people’s attitudes toward HPV vaccination [14]. Cole-Lewis et al conducted a content analysis to identify key conversation trends about electronic cigarettes (e-cigarettes) by using historical Twitter data [15].

In this paper, we use Lynch syndrome as a case study to find popular Lynch syndrome–related topics discussed on Twitter, examine the correlations between promotional Lynch syndrome–related information (eg, information related to advertising, sales promotion, and public relations) and laypeople’s discussions (eg, comments toward health services, opinions to a policy, and self-expression of their feelings), and learn the influence of Lynch syndrome awareness events on laypeople’s discussions. Note that we classified the tweets based on information types rather than user types. It is possible that a layperson (eg, Lynch syndrome patient) who was well educated about the disease could also post tweets to promote awareness of and deliver knowledge on Lynch syndrome. Nevertheless, these tweets were categorized into promotional information in our study. Analyzing laypeople’s discussions on Twitter will be an extremely helpful tool to glean into laypeople’s awareness, perceptions, and attitudes toward Lynch syndrome and colorectal cancer for various stakeholders, including pharmaceutical companies, government agencies, health care organizations and professionals, and news media. For example, health advocacy groups can adjust their health communication strategies from learning the hot topics in laypeople’s discussions to optimize the dissemination of promotional health information. Through understanding how awareness events could impact laypeople’s perceptions and attitudes, health care organizations have the opportunity to estimate the influence of their promotional health events on laypeople’s behavior for future planning.

The central objective of our study was to understand how promotional Lynch syndrome–related health information impact laypeople’s discussions on Twitter. This study aims to answer the following research questions (RQ):

RQ1: What are the most discussed topics in Lynch syndrome–related tweets?

RQ2: How promotional Lynch syndrome–related information on Twitter affects laypeople’s discussions in terms of topic distributions?

RQ3: Do the Colon Cancer Awareness Month (March) and the Lynch Syndrome Awareness Day (March 22) have any impact on laypeople’s discussions on Twitter and their attitudes?

## Methods

### Data Analysis Overview

**Figure 1:**
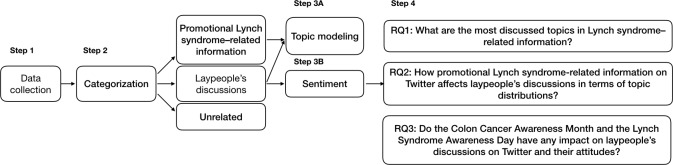
Twitter data processing and analysis workflow.

Our data analysis comprised the following 4 steps, schematized in Figure 1:

- Step 1 was data collection and preprocessing. We collected public tweets based on a set of keywords related to Lynch syndrome using the Twitter application programming interface (API). We then filtered out non-English tweets and standardized the texts (eg, hashtags and Web links).
- Step 2 was categorization of the tweets. We separated laypeople’s discussions from promotional Lynch syndrome–related information. We experimented with two methods to automatically classify the Twitter data—a convolutional neural network (CNN) and a rule-based classifier.
- Step 3 was topic modeling and sentiment analysis:
  - Topic modeling: We used the latent Dirichlet allocation (LDA) model to determine the major discussion themes in the collected Twitter dataset for both promotional information and laypeople’s discussions.
  - Sentiment analysis: We built a CNN to assign each tweet in the laypeople’s discussions with a sentiment label, namely, positive, negative, and neutral.
- Step 4 included RQs to examine the relationships between promotional Lynch syndrome–related information and laypeople’s discussions through analyzing the results of topic modeling and sentiment analysis. We presented frequency tables for Lynch syndrome–related topics on Twitter, correlations between promotional Lynch syndrome–related information and laypeople’s discussions, and trends of topics/sentiments in relation to awareness during the 2017 March Colon Cancer Awareness Month and the March 22 Lynch Syndrome Awareness Day.

Through these analyses, we aimed to answer the three RQs posted above.

### Step 1: Data Collection and Preprocessing

Tweets related to Lynch syndrome were collected from October 26, 2016 to August 11, 2017 (289 days) using a Twitter crawler [16] based on a set of keywords related to Lynch syndrome (ie, “lynch syndrome,” “#lynchsyndrome,” “lynchsyndrome,” and “#lynch_syndrome”), and non-English tweets were filtered out. To generate the list of keywords, we used a snowball sampling process. We started with a set of relevant seed keywords (eg, “lynch syndrome”). Then, we searched on Twitter with these keywords to retrieve a sample of tweets, evaluated whether the retrieved tweets were indeed relevant to Lynch syndrome, and identified additional keywords to be used for the next rounds of searches. The snowball sampling process was conducted iteratively until no new keyword was identified. We chose the specific time period (ie, from October 26, 2016 to August 11, 2017), as one of our RQs was to examine the impact of the awareness activities (ie, the 2017 March Colon Cancer Awareness Month and the March 22 Lynch Syndrome Awareness Day). This dataset gave us sufficient samples to compare the effects (eg, tweet volume changes, laypeople’s sentiment changes, and discussion topic changes) before, during, and after the events.

We then preprocessed the content of the tweets following the preprocessing steps used by GloVe [17] with minor modifications as follows: (1) all hashtags (eg, #Lynchsyndrome”) were replaced with “<hashtag> PHRASE” (eg, “<hashtag> lynchsyndrome”); (2) user mentions (eg, “@MyGeneCounsel”) were replaced with “<user>”; (3) Web addresses (eg, “https://t.co/fMmFWAHEuM”) were replaced with “<url>”; and (4) all emojis were replaced with “<emoji>.”

### Step 2: Categorization of Tweets

We used a two-step process to categorize the tweets into 3 categories (ie, unrelated, promotional Lynch syndrome–related information, and laypeople’s discussions). In the first step, we classified the tweets into related versus unrelated, whereas in the second step, the tweets were further classified into promotional Lynch syndrome–related information versus laypeople’s discussions. Due to the size of the dataset, it was not feasible to manually annotate all tweets. Thus, we explored two methods to build supervised models to automatically classify the collected tweets. We fitted a CNN classifier and built a simple rule-based classifier. We compared the performance of the two methods and used the model with the best performance balancing precision, recall, and F-measure.

#### A Convolutional Neural Network Classifier

CNNs have been widely used for sentence classification tasks with state-of-the-art performance [18]. To build a CNN classifier, we first needed an annotated training dataset. We randomly selected 1000 tweets, which were read and labeled by 2 reviewers independently. The annotation task was to categorize each tweet into one of the following three classes: (1) irrelevant to Lynch syndrome (ie, even though a tweet contains Lynch syndrome–related keywords, the tweet may not be relevant to Lynch syndrome, eg, “I don't have time or patience or business entertaining nobody Willie Lynch syndrome”); (2) Lynch syndrome–related promotional information (eg, news, research articles, promotional messages, and advertisements such as “RT @ShewithLynch: #Lynchsyndrome #News: Earlier Screening Could Save Many From Colorectal Cancer, Research Suggests - https://t.co/DVEb2xaD”); and (3) laypeople’s discussions related to Lynch syndrome (eg, “First #colonoscopy appointment set. I’ll have to do this at least once a year for the rest of my life #lynchsyndrome #coloncancerawareness”).

A common strategy for building CNN sentence classifiers is to use word embedding [19] to transform raw texts into vectors of real numbers as features. We used the pretrained word vectors from GloVe, which were trained on 2 billion tweets with 1.2 million vocabularies. For each tweet, a matrix was built by mapping each word in the tweet to its corresponding word embedding vector in d dimensions. As the length of each tweet varies, we padded tweets whose lengths were smaller than the longest tweet with zeros. Thus, all tweets were transformed into word embedding feature matrices with the same dimension. These feature matrices were then fed into the CNNs.

We built two CNNs using the same feature matrices: one that classified the tweets into relevant versus irrelevant, and another one that further classified the relevant tweets into promotion Lynch syndrome–related information versus laypeople’s discussions.

#### Rule-Based Classification

Through examining a random sample of the collected tweets, we found that 96% of the irrelevant tweets have the keywords “willie” or “willy,” referring to a person named “Willy Lynch.” Thus, we built a simple rule-based classifier that categorized a tweet as irrelevant if it contains any of the two keywords. Furthermore, within the relevant dataset, we observed that 88% of laypeople’s discussions did not contain any links. The promotional Lynch syndrome–related tweets were usually mentions of Lynch syndrome–related news, research findings such as new diagnostic or treatment techniques, and health promotion activities. Due to the 140-character length limit of each tweet, users often used hyperlinks in their tweets to refer to the source articles. On the contrary, laypeople’s discussions were typically expressions of their own attitudes or opinions without any references to other sources of information. Thus, in the second step, a tweet was classified as promotional Lynch syndrome–related information if the tweet contains any links. Otherwise, the tweet was categorized as a layperson’s discussion.

### Step 3A: Topic Modeling

In natural language processing, a topic model is a statistical model that can discover abstract topics in a collection of documents [20]. We used the LDA algorithm in this study to find main topics that are presented in the overall Twitter data, including both promotional Lynch syndrome–related information and laypeople’s discussions [21]. LDA is a generative model that represents each document (ie, a tweet in our case) as a mixture of latent topics, and each topic can generate words with certain probabilities. One of the most significant features of topic models is that they do not require any prior annotations or labeling of the documents. Nevertheless, similar to many other unsupervised clustering algorithms, the number of topics is a parameter that needs to be determined a priori. We experimented with three different statistical methods for finding the appropriate number of topics for LDA as follows: (1) Arun2010: Arun et al viewed LDA as a matrix factorization mechanism that can decompose a topic distribution into matrix factors. They then computed the symmetric Kullback-Leibler divergence of salient distributions that are derived from these matrix factors. They observed that the divergence values are higher for the nonoptimal number of topics [22]; (2) Cao2009: Cao et al considered the LDA process similar to the density-based clustering algorithms. Thus, the goal of finding the best number of topics is similar to finding the best number of clusters, where it maximizes the intracluster similarities while minimizing the intercluster similarities [23]; and (3) Deveaud2014: Deveaud et al, similar to the Arun2010 method, used a simple heuristic that estimates the number of latent topics by maximizing the information divergence (ie, Jensen-Shannon divergence) among all pairs of LDA’s topics [24]. However, these statistical methods do not always converge, and often, the number of topics discovered does not conform to human judgments. Thus, additional qualitative analysis of the generated topics to determine their quality is still necessary.

Before applying the LDA algorithm, we further preprocessed the Twitter data to lemmatize the words and to remove words that are commonly used but irrelevant to the topics that we aim to discover based on a list of stop words (eg, “it,” “he,” “she,” and “that”). We followed the best practices in training LDA models. As we learned probability distributions of words per topic (and a probability distribution of these topics over the entire collection of documents, ie, tweets) through LDA, each topic can be naturally visualized as word clouds where the sizes of the words are proportional to their probabilities on the topic.

To learn the volume trend of each topic, we also need to know the topic of each tweet. An LDA model can also assign each tweet with topics based on the content of the tweet. As described in the LDA model, each tweet is a mixture of topics, where each topic has a certain probability to appear in the tweet. Thus, all topics have a probability value for each tweet, and topics that are unlikely to appear have a small probability value. In other words, each topic assigned to a tweet has a probability to represent how a tweet will be classified into that specific topic. Thus, we needed to determine a cutoff for the topic probability values so that each tweet was assigned an accurate topic. In cases where the tweet was assigned more than one topics, we chose the topic with the highest probability value.

**Three research questions analyzed using the results of topic modeling and sentiment analysis to understand the impact of promotional Lynch syndrome–related information on laypeople’s discussions.**

1. What are the thematic topics in Lynch syndrome–related tweets?
  - We qualitatively analyzed the topics discovered from the latent Dirichlet allocation (LDA) model and visualized the latent topics with a set of word clouds.
  - We plotted the volume of tweets for each topic category and ranked the topics by popularity.
  - We examined the descriptive statistics of the overall laypeople’s sentiments as well as their sentiments by topic.
2. How promotional Lynch syndrome–related information on Twitter affects laypeople’s discussions in terms of topic distributions?
  - We calculated the proportion of each topic within their user groups (ie, promotional Lynch syndrome–related information and laypeople’s discussions) and visualized the topic distribution results as word clouds to examine whether promotional Lynch syndrome–related information has a similar topic distribution to laypeople’s discussions.
  - We plotted the monthly trends of the topics for both promotional Lynch syndrome–related information and laypeople’s discussions. We also examined the correlations between these trends using the Pearson correlation efficient.
3. Do Colon Cancer Awareness Month (March) and Lynch Syndrome Awareness Day (March 22) have any impact on laypeople’s discussions on Twitter and their attitudes (ie, positive, negative, and neutral)?
  - We examined how the overall tweet volume changed during these time periods as well as how the tweet volumes of different topics changed.
  - We also plotted the trends of people’s overall sentiments and their sentiments by topic across the entire time period and examined the changes during the event times.

### Step 3B: Sentiment Analysis

Sentiment analysis is a popular natural language processing method frequently used to determine the opinion, attitude, or the emotional state of the writer from a piece of writing. A basic task in sentiment analysis is to classify the polarity (ie, positive, negative, and neutral) of a given text. There are two main sentiment analysis approaches [25] as follows: (1) machine learning–based methods that build classification models from labeled training data and (2) lexicon-based techniques such as Linguistic Inquiry and Word Count [26] that ties word choices to authors’ opinions. Following the machine learning–based approach, we built a CNN model following the same process we used in step 2 for sentiment classification. The training data contained 1092 tweets (ie, we started with 1500 random tweets; after deduplication, 1092 tweets were left for annotation) randomly selected from laypeople’s tweets (ie, as we were only interested in laypeople’s attitudes toward Lynch syndrome) and annotated by two coders (YZ and LJRD) into three categories: positive, negative, and neutral. The Cohen kappa is .89, which suggests a strong agreement between the two coders. A third reviewer (JB) was consulted to resolve the disagreements between the two coders.

### Step 4: Research Questions

We answered the three RQs through analyzing the results of topic modeling and sentiment analysis in the following steps (Textbox 1).

## Results

### Step 1: Data Collection and Preprocessing

**Figure 2:**
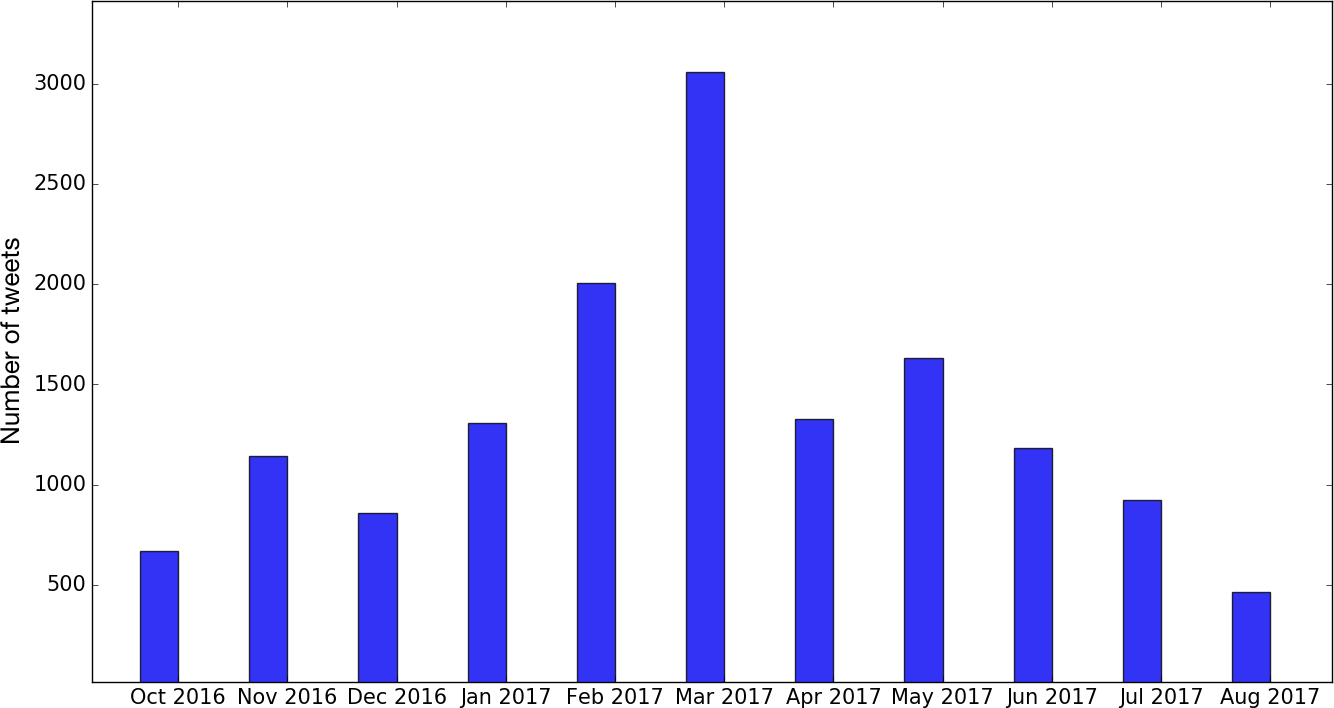
The number of English tweets collected with Lynch syndrome–related keywords by month.

Using the Twitter API via a Twitter crawler [16], a total of 16,667 tweets were collected from October 26, 2016 to August 11, 2017. After preprocessing and removal of non-English tweets, there were 14,564 tweets left. Figure 2 shows the monthly distribution of the English tweets during that time period.

### Step 2: Categorization of Tweets

The annotation task created a gold standard dataset of 1000 random tweets. There was a moderate agreement between the two coders (ie, Cohen kappa=.72) [27]. A third person reviewed the disagreements and placed those tweets into the appropriate category. We explored two classification methods (ie, a CNN model and a rule-based classifier) and compared their performance. As the dataset is unbalanced (ie, most tweets were relevant as we used very specific keywords to collect these data, and there were more promotional tweets than laypeople’s discussions), we used the weighted precision, recall, and F-measure to measure the classifiers’ performance.

As shown in Table 1, even though the rule-based classifier is simple, it outperformed the complex CNN model in both classification tasks. Thus, we used the rule-based classifier to categorize all tweets. Out of the 14,564 English tweets, 11,860 tweets were classified as relevant. Within the relevant tweets, 2705 tweets belonged to laypeople’s discussions, and 11,077 were promotional Lynch syndrome–related information.

**Table 1:** A comparison of the two classifiers’ performance.

Classification Methods	Relevant versus irrelevant			Promotional versus laypeople		
	Precision	Recall	F-measure	Precision	Recall	F-measure
Convolutional neural network	.651	.807	.720	.514	.717	.599
Rule-based	.938	.935	.936	.877	.870	.873

### Step 3A: Topic Modeling

**Figure 3:**
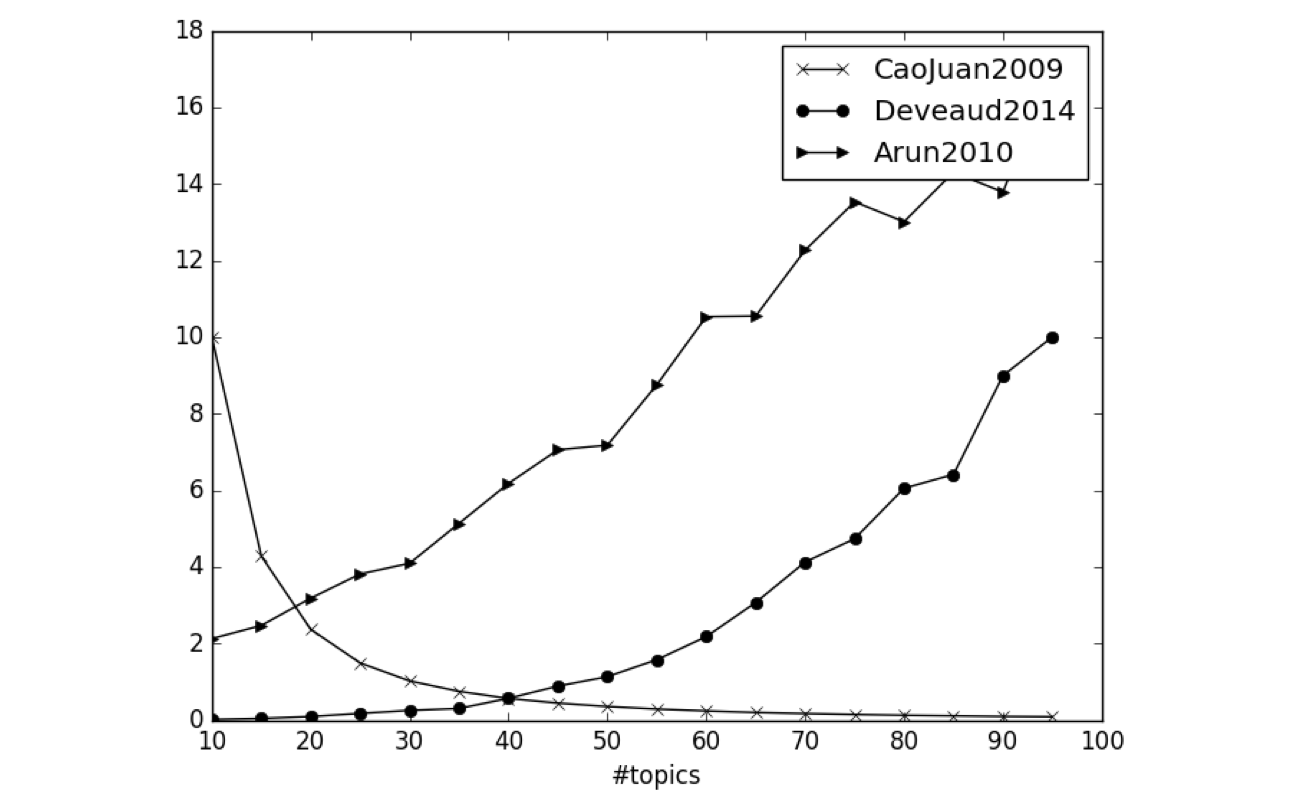
The three topic modeling quality measures by the number of topics.

We tried all three statistical methods to find the number of topics in the Lynch syndrome–related tweets (ie, tweets that were classified as relevant). As shown in Figure 3, none of the three methods converged and allowed us to select the appropriate number of topics. Note that we did not show the units of the y-axis in Figure 3 as the three different measures have different units. Nevertheless, the units of the measures were not important as the goal was to find the “elbow” points of the curves, which would indicate the optimal number of topics.

**Figure 4:**
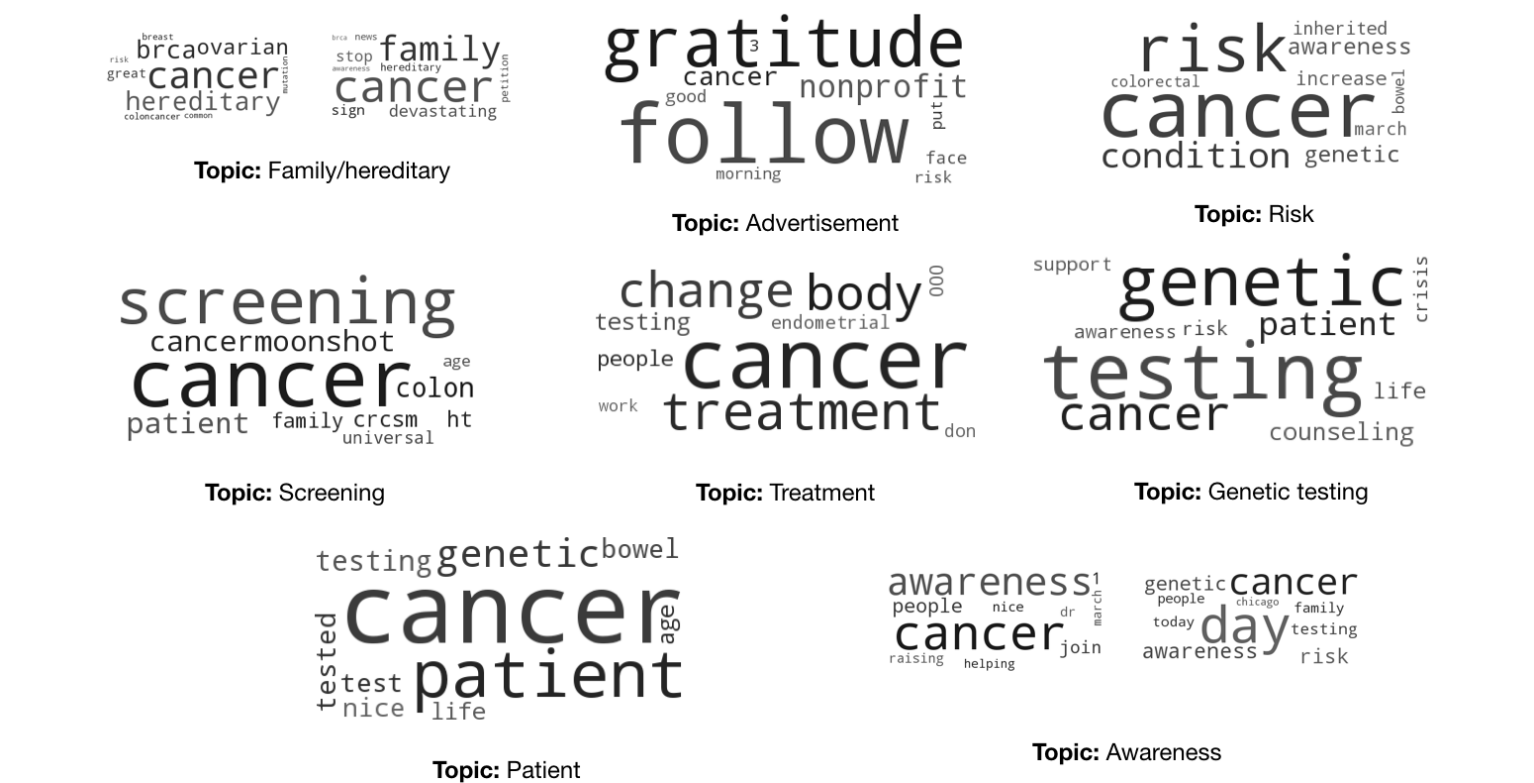
The eight topics learned from Lynch syndrome–related tweets.

Thus, we experimented with 10, 15, 20, and 30 topics and used word clouds to visualize the results. In each iteration, varying the number of topics (K=10, 15, 20, and 30), two coders were presented with the word clouds and a set of example tweets of the topics and were asked to assign each topic a label based on their judgments, independently. Each coder was also asked to identify duplicate topics and topics with poor quality (ie, the keywords in the topic did not represent a cohesive concept). We then chose a *K* that generated the least number of duplicate topics and inadequate topics. We determined that the most adequate number of topics was 10 and identified the labels for all topics. In cases where the coders did not agree on the particular label, the conflicts were resolved through discussions with the entire study team. We also merged the topics that had similar semantics into a single category. For example, “awareness event” typically contains event information to raise Lynch syndrome awareness, whereas the tweets in the “awareness” theme raise Lynch syndrome knowledge. We, thus, combined “awareness event” and “awareness” to “awareness/awareness event.” The final extracted topics and associated word clouds are shown in Figure 4.

After generating the topics, the LDA model was also able to assign a topic probability distribution for each tweet. As shown in Table 2, LDA assigned a probability value to every topic, even when a topic is unlikely to be in the tweet. Thus, we needed to find a cut-off probability value to extract the main topics of each tweet. We first generated a random sample of 50 tweets and iteratively tested different cut-off values. In each iteration, we evaluated the topics above the cut-off probability value assigned to the 50 tweets and manually determined whether the assignments were appropriate. We chose the cut-off value that generated the minimum number of topics for each tweet, and the accuracy was above 80% (ie, more than 80% of the tweets had the correct topic assignments through manual review). As a result, some tweets were assigned multiple topics, whereas others did not have any topics. Table 3 shows an example of tweets in each topic.

**Table 2:** Example of topics and their probabilities assigned to each tweet.

Category	Tweet	Top 3 topics (topic probability)
Promotional	“What is risk of pts w #Lynchsyndrome developing various cancers over time? Population-based study offers answers.”	Risk (.644), genetic testing (.197), treatment (.118)
	“Adapting to body changes during #cancer treatment #LynchSyndrome”	Treatment (.533), patient (.276), family (.139)
Laypeople	“I have Lynch Syndrome with 60-80% chance of dying from colon cancer just like my mother and brother #IAmAPreexistingCondition”	Family and hereditary (.442), screening (.327), patient (.172)
	“My #breastcancer diagnosis caused me to get a #genetics test & found out I have a gene 4 #LynchSyndrome #earlydetection #ColonCancerMonth”	Patient (.716), risk (.128), awareness/awareness event (.119)

**Table 3:** Example tweets by topic.

Topics	Example Tweets
Family and hereditary	“This week, we highlight Lynch Syndrome, Familial Hypercholesterolemia & Hereditary Breast & Ovarian Cancer.”
	“Aiming to prevent hereditary cancers, researchers focus on #LynchSyndrome #NCICancerCurrentsBlog #Cancer”
Screening	“#Lynchsyndrome #News: Earlier Screening Could Save Many From Colorectal Cancer, Research Suggests”
	“Universal tumor screening for #Lynchsyndrome: health-care providers’ perspectives.”
Advertisement	“Gratitude to our new followers! Join us #Monday for #GenCSM! #Lynchsyndrome #HereditaryColorectalCancer”
	“#Lynchsyndrome #GenCSM: Gratitude to all of my new followers! Have a stellar day!! G @ the #Nonprofit:”
Treatment	“Total abdominal colectomy is recommended for treatment of CRC in individuals who are known to have #LynchSyndrome #Hered,”
	“#Treatment Continues to Advance in #OvarianCancer and Other Gynecologic Malignancies”
Patient	“Patient with newly found #LynchSyndrome says 30+yo children refuse testing due to ‘inconvenience’.” Hope time/education change minds #GCchat,”
	“1/44 #coloncancer patients have #Lynchsyndrome @HHampel1 @theNCI #Moonshot #hereditarycancer”
Risk	“btw, glioblastoma is very malignant + chemicals like pesticides are risk factors. Genetic disorders like Lynch syndrome is a risk factor.”
	“Authors state that the cumulative lifetime risk to develop ovarian cancer in their patients with Lynch syndrome: 20% by age 80”
Genetic testing	“mom got back the genetic tests and apparently they pinged the tumor to a genetic mutation so 24% chance of her having lynch syndrome ;;; ugh”
	“Inherited colon cancer syndromes can be predicted through genetic testing. #GetScreened #LynchSyndrome”
Awareness/awareness event	“Happy #lynchsyndromeawarenessday! #Lynchsyndrome #Genetics”
	“#coloncancer awareness month - if U were diagnosed w/ CRC, make sure your tumor was screened 4 #Lynch syndrome with IHC or MSI testing”

### Step 3B: Sentiment Analysis

We trained a sentiment CNN classifier with the 1092 annotated tweets. We followed the best practices in machine learning experiments to build the CNN, for example, use 80% of the tweets as the training dataset, and measured the performance of the classifier on the remaining 20% hold-out test set. The performance of the CNN classifier was reasonable (ie, precision: .737, recall: .766, F-measure: .736, and accuracy: .766).

### Step 4: Research Questions

#### RQ1: What Are the Thematic Topics in Lynch Syndrome–Related Tweets?

**Figure 5:**
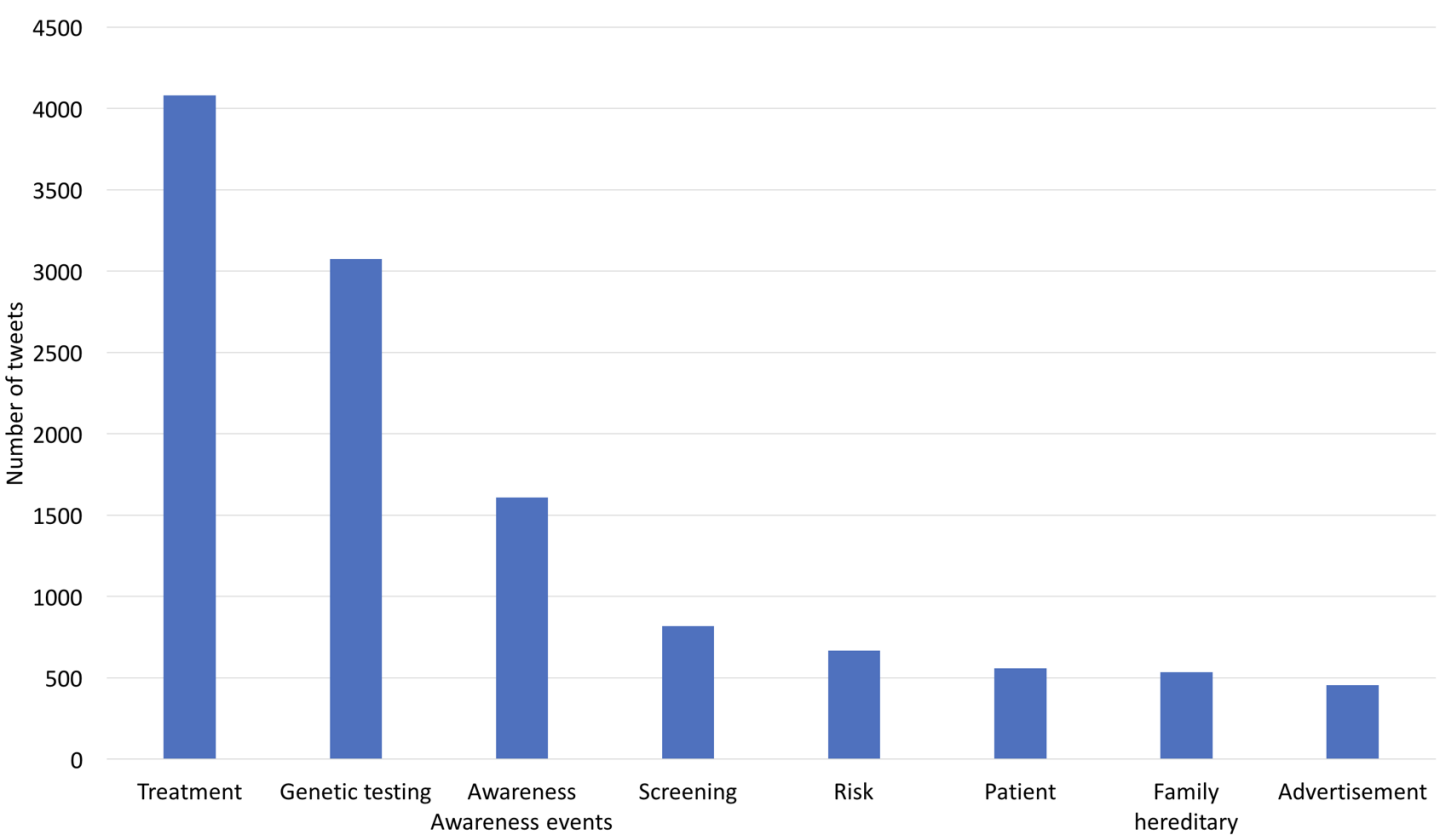
The number of tweets across different topics learned from the Latent Dirichlet allocation model.

We plotted a histogram of tweet volumes by topic and ranked the topics by volume as shown in Figure 5.

*Treatment*, *genetic testing*, and *awareness* were the top three topics in Lynch syndrome–related tweets.

We plotted the sentiment distribution of the overall laypeople’s discussion tweets as well as the sentiment distribution of each topic as shown in Table 4. Overall, most of the tweets were neutral (78.07%), although there were significantly more positive (18.42%) than negative (3.51%) tweets. Across the sentiment distribution of topics, only the *treatment* topic had more negative (16.67%) than positive tweets.

#### RQ 2: How Promotional Lynch Syndrome–Related Information on Twitter Affects Laypeople’s Discussions in Terms of Topic Distributions?

**Figure 6:**
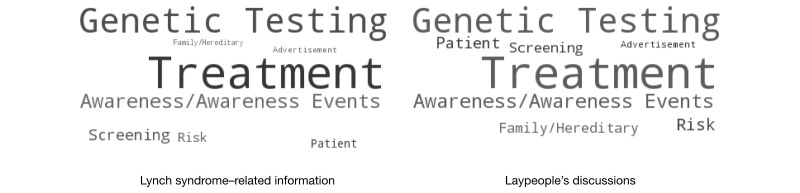
Topic proportions of promotional Lynch syndrome–related information and laypeople’s discussions.

We calculated the proportion of each topic in both promotional Lynch syndrome–related information and laypeople’s discussions and visually compared the results by using word clouds. As shown in Figure 6, the topics and their proportions in the laypeople’s discussions were similar to those in the promotional Lynch syndrome–related information.

**Figure 7:**
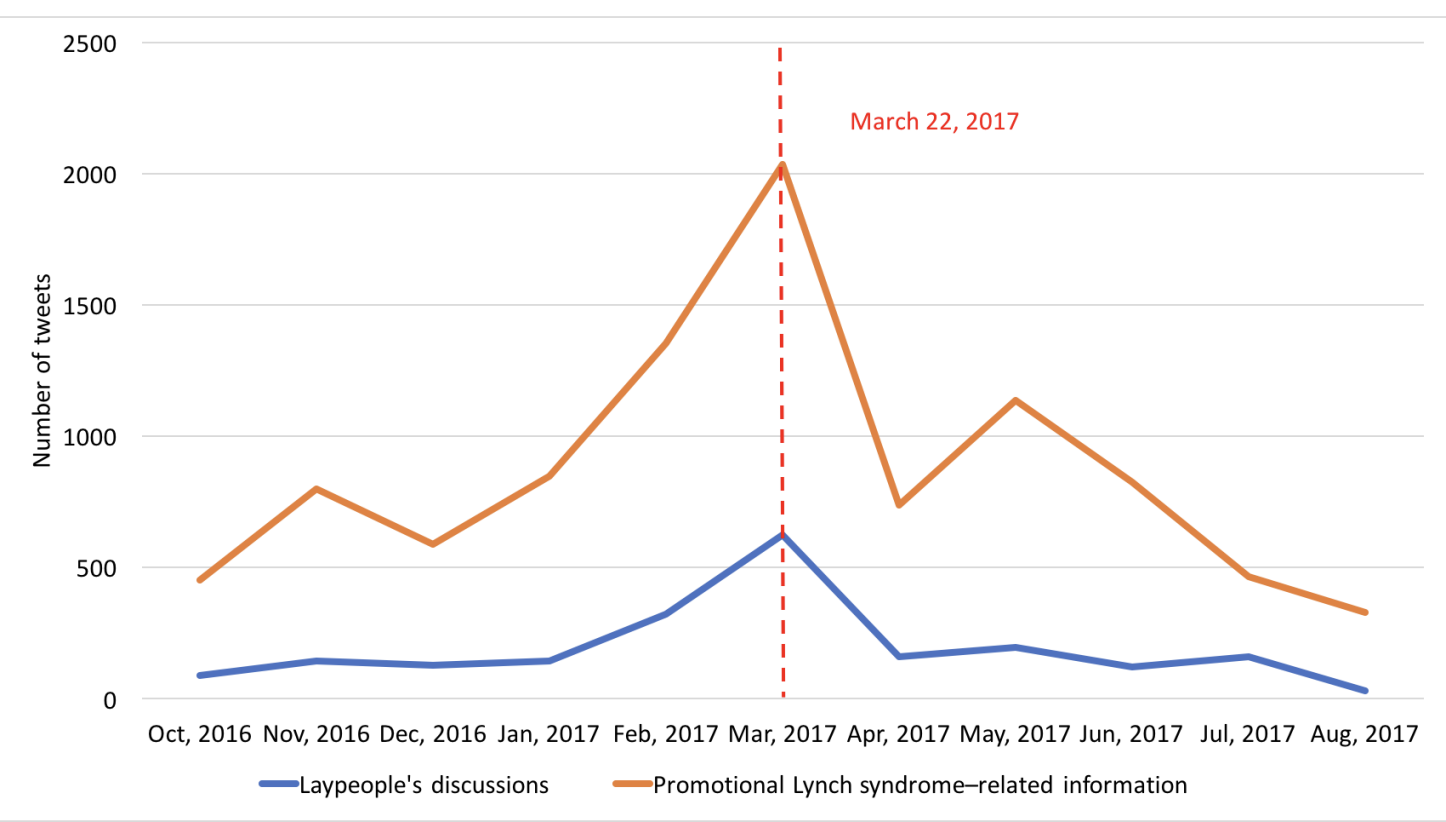
The number of Lynch syndrome–related tweets by month and by tweet type (ie, promotional Lynch syndrome–related information vs laypeople’s discussions).

We also calculated the Pearson correlation coefficient [28] between the promotional Lynch syndrome–related information and laypeople’s discussion based on their monthly tweet volumes (as shown in Figure 7). As shown in Table 5, laypeople’s discussions had a strong correlation with promotional Lynch syndrome–related information on the *awareness* topic and moderate correlations on the topics *screening*, *genetic testing*, *treatment*, and *risk*.

**Table 4:** Laypeople’s overall sentiment distribution on Lynch syndrome and their sentiment distributions across topics.

Topic	Positive (%)	Negative (%)	Neutral (%)
Family and hereditary	31 (35.63)	2 (2.30)	54 (62.07)
Screening	11 (8.73)	3 (2.38)	112 (88.89)
Advertisement	36 (41.86)	2 (2.33)	48 (55.81)
Treatment	0 (0.00)	78 (16.67)	390 (83.33)
Patient	97 (49.75)	1 (0.51)	98 (49.75)
Risk	24 (12.00)	0 (0.00)	176 (98.00)
Genetic testing	28 (17.40)	9 (5.59)	124 (77.00)
Awareness and awareness events	60 (20.00)	0 (0.00)	240 (80.00)
Overall	498 (18.42)	95 (3.51)	2111 (78.07)

**Table 5:** Pearson correlation coefficients between promotional Lynch syndrome–related information and laypeople’s discussions based on their monthly tweet volumes.

Topic	Correlation coefficient	*P* value
Family/hereditary	.479	.14
Screening	.602	.05
Advertisement	.112	.74
Treatment	.698	.02
Patient	.211	.53
Risk	.659	.03
Genetic testing	.624	.04
Awareness/awareness events	.989	<.001

#### RQ 3: Do the Colon Cancer Awareness Month (March) and the Lynch Syndrome Awareness Day (March 22) Have Any Impact on Laypeople’s Discussions on Twitter and Their Attitudes (ie, Positive, Negative, and Neutral)?

**Figure 8:**
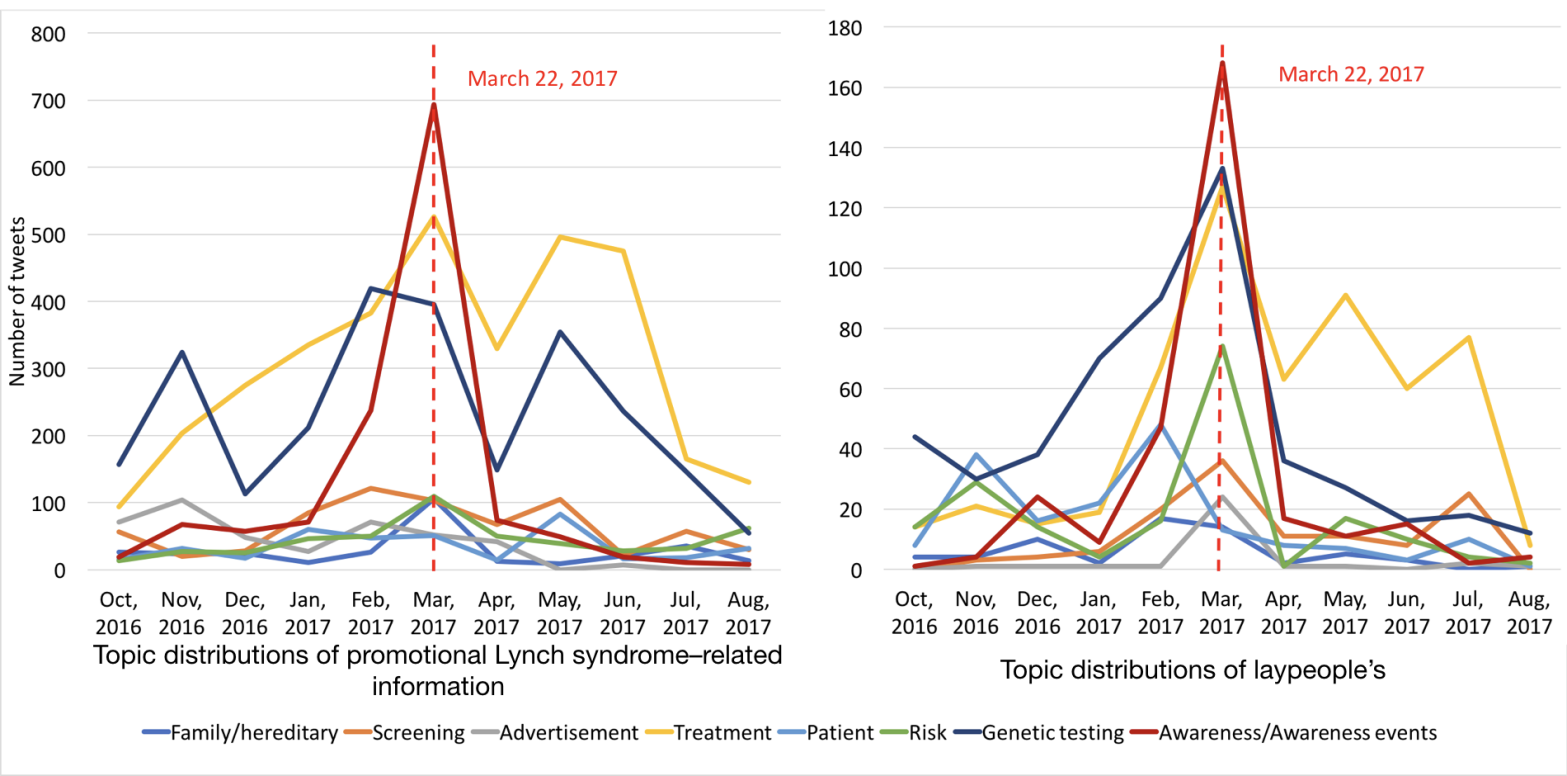
The number of Lynch syndrome–related tweets by month and by topic.

As shown in Figure 7, the overall tweet volume increased dramatically during the March Colon Cancer Awareness Month and peaked around the Lynch Syndrome Awareness Day on March 22, 2017. Furthermore, as shown in Figure 8, the tweet volumes of individual topics followed the same pattern, especially for “awareness/awareness events,” “genetic testing,” and “patient.”

**Figure 9:**
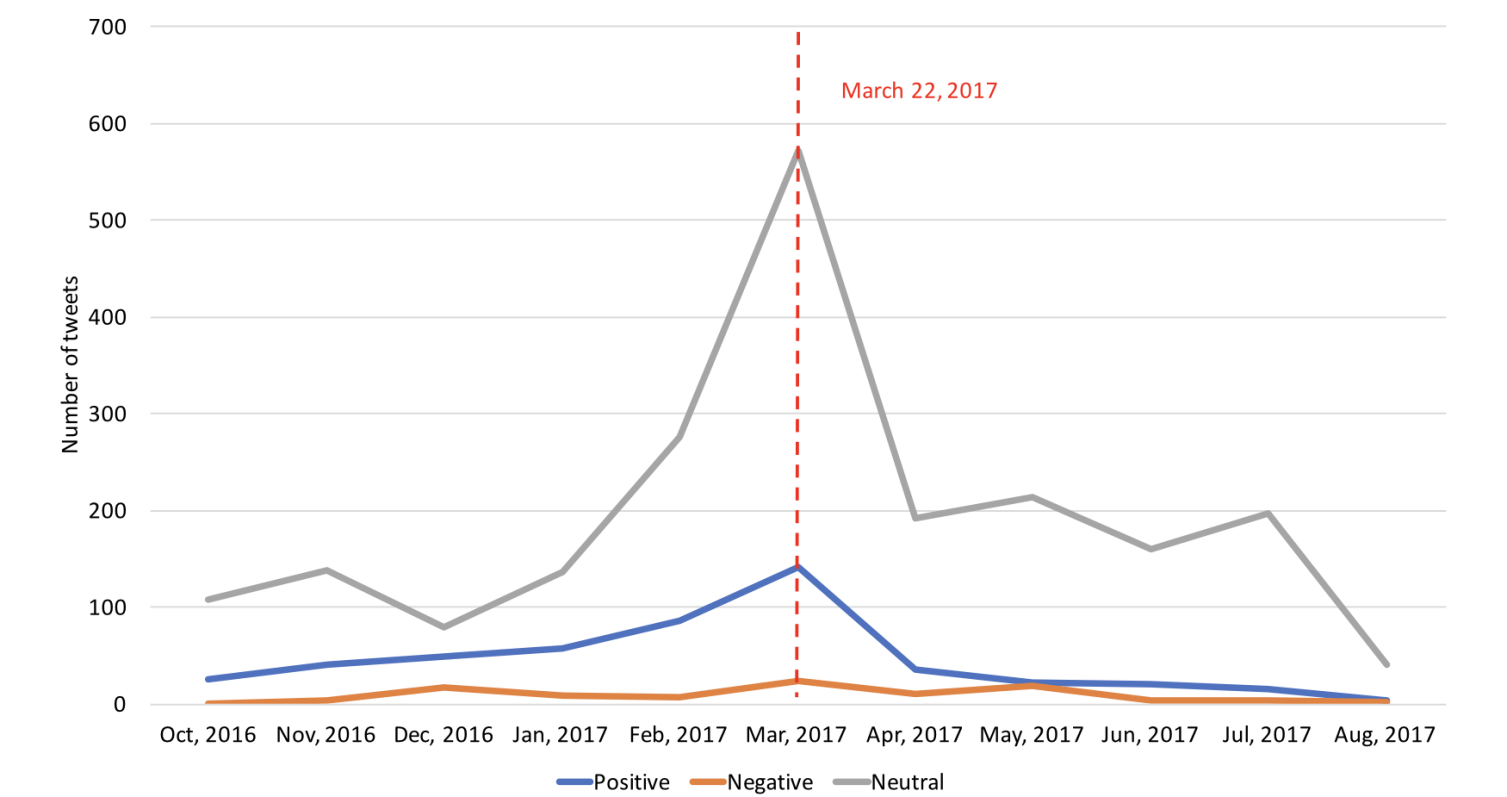
The number of tweets by month and by laypeople’s sentiment.

We then plotted the overall tweet volume trends by different sentiment categories in laypeople’s discussions as shown in Figure 9. The volume of negative tweets remained roughly the same across the entire time period. The volume of neutral tweets sharply increased in the month of March, reflecting a significant tweet volume increase during that month (Figure 2). The volume of positive tweets also increased in March, but it was less aggressive than neutral tweets.

**Figure 10:**
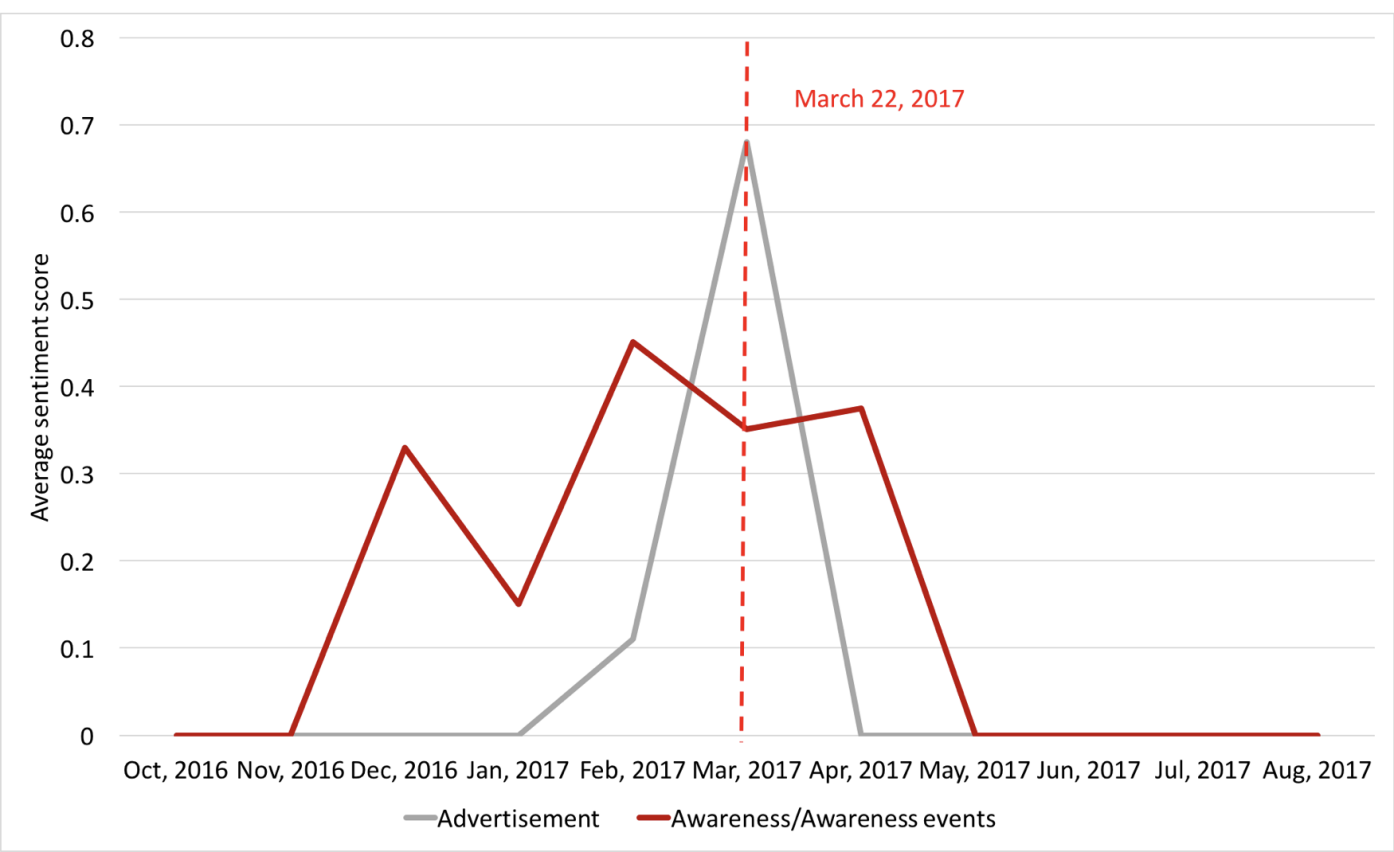
The average sentiment scores for “advertisement” and “awareness/awareness events” topics by month.

We further analyzed laypeople’s sentiment trends by topic to understand on which topics the laypeople had obvious attitude changes during the awareness events. We constructed an average sentiment score for each month for each topic. For each individual tweet, we assigned it a score of 1 if it was positive, 0 if it was neutral, and −1 if it was negative. We summed up the scores for all tweets in each topic by month and normalized the score by the total number of tweets in that topic category for that month. As shown in Figure 10, the average sentiment scores for “advertisement” and “awareness/awareness events” increased significantly during the March Awareness Month but dropped immediately afterward. There were no clear sentiment trends for other topics.

## Discussion

### Principal Findings

The goal of our study was to understand how promotional Lynch syndrome–related health information impacts laypeople’s discussions on Twitter. We used topic modeling and sentiment analysis on Lynch syndrome–related tweets to answer the following 3 RQs: (1) what are the most discussed topics in Lynch syndrome–related tweets?; (2) how promotional Lynch syndrome–related information on Twitter affects laypeople’s discussions?; and (3) what impact do the Lynch syndrome awareness activities in the Colon Cancer Awareness Month and Lynch Syndrome Awareness Day have on laypeople’s discussions and their attitudes? We found that “awareness,” “treatment,” and “genetic testing” were the most popular topics in Lynch syndrome–related tweets. Furthermore, laypeople’s attitudes toward “treatment” and “genetic testing” were relatively negative compared with other topics they discussed on social media. It is not surprising that most of the information related to Lynch syndrome on Twitter focused on treatment and genetic testing, and people had more negative attitudes toward these topics because they feared the possibility of having a higher cancer risk or a positive cancer diagnosis and worried about the costs and the quality of the diagnostic methods (eg, “I have had Cancer twice fear of 3x is always on my mind. Not having Medicare is heartbreaking for me” and “cost of genetic testing for lynch syndrome mercedes 300se”).

The topic distributions of promotional Lynch syndrome–related information and laypeople’s discussions were similar. Especially, laypeople’s discussions on “awareness” were highly correlated with the promotional Lynch syndrome–related information on Twitter, whereas their discussions on “screening,” “genetic testing,” “treatment,” and “risk” were moderately correlated. These results suggest that the promotional information posted by health care organizations and professionals on social media platforms such as Twitter may have a significant impact on laypeople. In part, our results provided the evidence to support the rationale for further developing novel cancer communication strategies in new digital media [29].

Furthermore, health-related awareness events and initiatives such as the March Colon Cancer Awareness Month and the March 22 Lynch Syndrome Awareness Day have great impacts on laypeople’s discussions, perceptions, and attitudes of the health condition. Our analysis of the monthly tweet volume trends revealed that health organizations and professionals made a concerted effort to disseminate promotional Lynch syndrome–related information on Twitter during these awareness events. Furthermore, their efforts had a great impact on raising laypeople’s awareness of the specific health topic, which was evident from the increased tweet volume by laypeople during these awareness events. Moreover, we also observed that laypeople had more positive attitudes during these events as shown in Figure 10. Interestingly, laypeople’s attitudes toward certain topics such as “advertisement” and “awareness/awareness events” became more positive than other topics during these awareness events. The changes in attitudes may be explained by the theory of social influence [30,31]. As laypeople received more positive information about colorectal cancer and Lynch syndrome, they gained a better understanding of the health condition and perceived better health outcomes, which could lead to more positive thinking.

The possibility to positively influence laypeople’s attitudes and their normative beliefs toward Lynch syndrome gives us the opportunity to design novel participative communication strategies in cancer prevention and control in accordance with behavior change theories. For example, in the theory of planned behavior [32], both attitudes and normative beliefs can shape an individual’s behavioral intentions and behaviors. Nevertheless, evident from our results, as shown in Figures 7-10, both the volume of the Lynch syndrome–related tweets and the positive sentiments of the laypeople dropped after the awareness events. These results suggest that these awareness events may need to be hosted frequently to have a sustained effect.

Designing an appropriate promotion strategy on social media needs more considerations than traditional media (eg, newspapers, television advertisements, and flyers). Health organizations and professionals need to think about what kind of information social media can deliver, and how the promotional information can achieve their goals (eg, enhancing communication with audience to foster public engagement). Many of the promotional Lynch syndrome–related information in our dataset indeed followed the recommendations for developing health promotion messages on social media [33-35], especially on disseminating critical health information (eg, sharing news, research findings, and the basic knowledge of Lynch syndrome) and engaging the public (eg, Colon Cancer Awareness Month and Lynch Syndrome Awareness Day).

As evidenced in our study, the use of social media is expanding rapidly in health promotions. It is increasingly important to measure the performance of these health promotion strategies. Neiger et al proposed a set of key performance indicators (KPIs) and metrics for evaluating the performance of health promotions in social media [35]. There are four indicators in the KPIs as follows: (1) insight (eg, consumer feedback from social media), (2) exposure (eg, the number of times a promotional information is viewed), (3) reach (eg, the number of people who have viewed the promotional materials and the related content), and (4) engagement (eg, “likes” on the posts, sharing and retweeting the posts, and engaging in the offline events). Our study results can provide more in-depth insights to many of these key indicators. For example, the sentiment analysis results will provide more fine-grained information on users’ attitudes toward these health promotion events than simple “likes” on the posts.

Our study focused on analyzing the texts of Lynch syndrome–related tweets, whereas Twitter collects much more information on both the tweets (eg, the links between tweets through retweeting) and their users (eg, user locations, friends, and followers). This information can be leveraged to conduct more in-depth analyses of health-related topics on Twitter. For example, through modeling the retweet networks, we can study how promotional health information spread on Twitter through social network analyses.

### Limitations

First, to automatically categorize tweets and assign each tweet a sentiment, we employed computational classification methods, whose accuracies were not perfect. This imperfection left the possibility of having incorrect results on a micro scale (ie, on individual tweets). Nevertheless, given the large volume of our data, the results on a macro scale should be consistent. Furthermore, we classified the tweets into promotional Lynch syndrome–related information and laypeople’s discussions. However, some of the tweets that we classified as laypeople’s discussions might be from health professionals and health advocacy groups. One way to alleviate this issue is to identify these users based on their Twitter user profiles and classify their tweets accordingly. Moreover, the demographics (eg, age, gender, race, and ethnicity) of Twitter users might be confounding variables in our analyses that might need to be controlled. Nevertheless, there was not an easy way to identify Twitter users’ demographics, as Twitter does not require its users to provide such information.

Second, topic modeling can only extract abstract topics at a high level. These abstract topics often had more in-depth aspects to explore. For example, “genetic testing” can be further divided into more fine-grained aspects (eg, cost of genetic testing and accuracy of genetic testing). One way to address this issue is to develop a coding book and manually annotate each individual tweet with the fine-grained topics. Nevertheless, such process is labor-intensive and hardly possible with a large volume of Twitter data. One possible solution is to label a small random sample of the tweets and then develop supervised classifiers (similar to the approach we used for sentiment analysis) to label the rest of the data automatically.

Third, Twitter users are not a representative group of the general population. The majority of social media users, in general, tend to be younger; 71% of Twitter users in 2017 are less than 49 years old [36].

### Comparison With Prior Work

A number of studies have used sentiment analysis and topic modeling to analyze social media data on health-related topics. Doing-Harris et al designed a topic classifier and identified common topics on patient comments to understand patient satisfaction toward health services [37]. Lu et al determined the hot topics and measured sentiment expression of different stakeholders to understand their different perspectives [38]. Guillory et al used Twitter data to analyze e-cigarette discussions based on discussion theme and sentiment [39]. Wang et al used keywords matching and topic modeling as well as qualitative methods on social media data to learn actionable information about pollution levels and public responses [40]. Davis et al applied sentiment analysis on Twitter data to learn the public’s response to Obamacare [41]. To our knowledge, our study is the first on using Twitter data to understand the correlation between promotional health-related information and laypeople’s discussions.

### Conclusions

Our results provided evidence to confirm the positive impacts of awareness initiatives and events that have been widely promoted by health organizations and professionals on social media platforms. Furthermore, a deeper understanding of how these promotional information and events affect individuals’ attitudes and their perceived social norm could lead us to better-designed health behavior interventions. A number of future directions can further advance our understanding of the impacts of promotional information on laypeople. For example, it will provide additional context and information through examining the Twitter users’ profiles and the sources of the promotional materials (following the links in the tweets). Nevertheless, more advanced natural language processing tools and machine learning models need to be developed to process the large amount of Twitter data.

## Abbreviations

API: application programming interface
CNN: convolutional neural network
HNPCC: hereditary nonpolyposis colorectal cancer
HPV: human papillomavirus
KPI: key performance indicators
LDA: latent Dirichlet allocation
RQ: research questions
